# Drivers and group analysis of governance efficiency of eco-environmental government-enterprise co-operation in China

**DOI:** 10.3389/fpubh.2025.1611688

**Published:** 2025-09-03

**Authors:** Xiaohu Yang, Xiaoling Yan, Guangyao Wang, Yanqin Wei, Meiling Zheng

**Affiliations:** ^1^School of Marxism, Shihezi University, Shihezi, China; ^2^Institute of Borderland Development and Governance, Shihezi University, Shihezi, China; ^3^School of Medical Administration, Nanjing Medical University, Nanjing, China; ^4^Laboratory for Digital Intelligence and Health Governance, Nanjing Medical University, Nanjing, China

**Keywords:** ecological environment, government-enterprise cooperation, governance efficiency, drivers, group analysis

## Abstract

The government-enterprise cooperation (GEC) model presents an innovative approach to China’s eco-environmental governance, effectively alleviating fiscal pressures on government, enhancing governance efficacy, and promoting balanced economic-ecological development across regions. This study employs the DEA method to measure eco-environmental GEC governance efficiency across 30 Chinese provinces (2010–2021), while utilizing the GTWR model to analyze spatiotemporal distribution patterns of efficiency drivers and their clustering characteristics. Key findings include: ① Temporally, China’s eco-environmental GEC governance efficiency demonstrates triphasic evolution: “declining-rising-declining.” ② Spatially, significant non-stationarity emerges with distinct high-low clustering patterns during the study period. ③ Driver analysis through spatiotemporal geographically weighted regression reveals substantial spatiotemporal heterogeneity in factor influences, with population density, technological investment, and innovation capacity emerging as key determinants through cluster analysis. Policy recommendations propose: (1) Implementing regionally differentiated GEC governance frameworks; (2) Enhancing technological sophistication and energy utilization efficiency in GEC systems; (3) Optimizing legal and market infrastructures for eco-environmental collaboration.

## 1 Introduction

China’s eco-environmental governance has long been plagued by systemic challenges including fragmented funding allocation [1.2 Trillion Dollar Fiscal Shortfall for Environmental Protection in 2021 Alone (1)], externalized governance benefits (2), and low economic return on investment (ROI) [The average profit margin of the environmental protection industry has long been 2.8 percentage points lower than that of the manufacturing industry (3)]. These structural deficiencies not only impede environmental protection progress but also constrain the sustainable development of related industries, as evidenced by the 43% underutilization rate of environmental protection facilities nationwide (4). While governmental intervention remains crucial, sole reliance on public-sector initiatives has proven inadequate—provincial environmental expenditure efficiency exhibits 58% regional disparity according to NBS 2022 data (5).

Recognizing these limitations, policymakers have actively sought institutional innovations through the “Modern Environmental Governance System” (2020) and market-oriented reforms articulated at the 2023 National Ecological Protection Conference (6). These initiatives emphasize public-private synergy through mechanisms like third-party governance and integrated resource markets for carbon/water/energy rights, with pilot programs achieving 37% cost reduction in Jiangsu’s industrial pollution control projects (7). Particularly, the government-enterprise co-operation (GEC) model emerges as a strategic response to dual pressures: alleviating fiscal burdens [The PPP model has attracted 2.1 trillion yuan of social capital to participate in ecological management (8)] while enhancing cross-regional ecological-economic coordination (9).

Nevertheless, three critical knowledge gaps persist. First, existing studies predominantly focus on governmental roles, neglecting systematic analysis of partnership dynamics under China’s unique market-socialism context. Second, the operational efficiency drivers of GEC remain underexplored, particularly regarding incentive alignment between environmental externalities and corporate profitability. Third, there lacks empirical evidence on how multi-stakeholder structures affect governance pathway selection across diverse regional ecosystems, as regional GEC performance varies by 2.9 times between eastern and western provinces. The identification of these three knowledge gaps is important because they limit the improvement of ecological environment governance efficiency, especially the inefficiency caused by insufficient governance by a single entity.

This study bridges these gaps through three original contributions:

(1) Developing an efficiency evaluation framework incorporating both ecological metrics and institutional transaction costs.
(2) Identifying key drivers through comparative analysis of 31 provincial GEC implementations (2010–2021)
(3) Proposing context-specific governance pathways based on regional economic-ecological typologies.

The findings provide policymakers with evidence-based strategies to optimize partnership models, while advancing theoretical understanding of collaborative environmental governance in transitional economies. As China’s experience offers paradigmatic insights for developing nations balancing environmental protection and economic growth, this research carries significant practical and academic implications.

## 2 Literature review and theoretical framework

### 2.1 Literature review

The evolution of eco-environmental governance research reflects methodological advancements across three paradigms: institutional analysis, efficiency quantification, and spatiotemporal dynamics. Early foundational studies established governance frameworks under China’s unique political ecology (10), while recent works increasingly adopt computational social science approaches to address system complexity (11).

#### 2.1.1 Methodological progression in efficiency evaluation

The application of Data Envelopment Analysis (DEA) marked a pivotal shift toward quantitative governance assessment. Dong Xiuhai et al. employed the DEA methodology to demonstrate suboptimal eco-environmental governance efficiency in China, advocating for enhanced pollution control and environmental infrastructure outputs (12). Zhang Qingmin et al. conducted an empirical analysis using a three-phase DEA model, revealing persistent optimization potential in China’s urban agglomeration governance efficiency (13). Beyond conventional DEA applications, Golany addressed unintended outputs through nonlinear functional approaches, though this methodological framework encounters limitations in accommodating data convexity (14). Tone’s SBM model advanced efficiency measurement by incorporating both radial and angular dimensions while accounting for input/output slack variables (15). Xu Chenglong et al. utilized SE-DEA and EViews 6.0 to identify key strategies for Shandong Province: developing circular economies, tightening environ-mental regulations, and implementing region-specific policies (16). Li et al. used a super SBM (based on relaxed measurement) model based on a panel Tobit regression model to explore the tourism ecological efficiency of 13 cities in the Beijing-Tianjin-Hebei region of China from 2010 to 2019 (17).

#### 2.1.2 Spatial–temporal analytical frontiers

While conventional regression dominates policy analysis (82% of sampled studies) (14), spatial econometrics has gained traction in infrastructure-impact studies. Notably, Liu et al. (18) in Transport Policy demonstrated railway-induced economic spillovers using spatial Durbin models, establishing methodological precedents for handling geographic heterogeneity. Wang et al. used a super-slack variable (SBM) model to calculate the ecological efficiency of tourism in Inner Mongolia from 2009 to 2019 and analyzed the factors influencing spatial evolution using a geographically weighted regression method (19). Hu et al. used a geographically weighted regression (GWR) model to assess regional differences in the impact of air pollutants on life expectancy (20). However, their focus on economic externalities differs fundamentally from environmental governance systems requiring spatiotemporal coordination of ecological thresholds (21). Parallel advancements in temporal granularity emerge through panel vector autoregression (PVAR) applications (22), yet integrated spacetime modeling remains scarce—only 12% of Web of Science-indexed environmental studies (2019–2023) adopted GTWR methods (23).

#### 2.1.3 Institutional innovation in collaborative governance

The government-enterprise partnership (GEC) paradigm has drawn increasing attention since China’s 2020 Modern Environmental Governance System reform. While Bao and Guan (24) identified fiscal mediation effects through regulatory channels, recent experimental evidence from Jiangsu’s PPP pilots shows 23–37% efficiency gains compared to state-led projects (25). Internationally, Marini et al. (26) market environmentalism theory provides conceptual scaffolding, though its Western-centric assumptions require adaptation to China’s socialist market context where administrative coordination remains pivotal (27).

#### 2.1.4 Critical research gaps

Synthesizing these developments reveals three unresolved challenges:

Temporal–spatial decoupling: Existing spatial analyses (21) predominantly examine cross-sectional patterns, neglecting the co-evolution of policies and ecosystems over time.

Partnership complexity: Current GEC studies (24–27) oversimplify principal-agent relationships, lacking empirical validation of incentive alignment mechanisms.

Driver interactivity: Conventional methods (DEA/PVAR/DID) examine linear causality, overlooking synergistic configurations between institutional, technological, and market factors.

#### 2.1.5 Methodological response

Our study advances an integrated analytical framework:

Spatiotemporal synthesis: Combining Geographically and Temporally Weighted Regression (GTWR) with ecological carrying capacity spatial weights, we extend Chen et al.’s (2023) spatial paradigm by incorporating dynamic policy-ecosystem interactions (2010–2021).

Hybrid causality: Embedding DEA-derived efficiency scores into fsQCA enables detection of nonlinear driver configurations—a critical advancement beyond single-dimension spatial effects.

### 2.2 Theoretical framework and methodological integration

Our methodological design is anchored in three-stage collaborative governance theory (28), which necessitates integrated assessment of governance efficiency, spatial–temporal drivers, and institutional configurations. This tripartite approach overcomes the “evaluation-identification gap” prevalent in environmental policy studies.

- Integration Rationale
- The methodological trilogy (DEA-GTWR-fsQCA) operationalizes three theoretical dimensions:
- Technical Efficiency (DEA): “How effective are GEC partnerships?”
- Dynamic Causality (GTWR): “Why does efficiency vary across space–time?”
- Institutional Synergy (fsQCA): “Which policy mixes optimize outcomes?”

This sequential logic mirrors the “diagnosis-prescription” framework in Social Cycle Theory (29), fulfilling both analytical and prescriptive research objectives. Recent applications in climate governance validate such hybrid approaches’ efficacy in addressing complex socio-ecological systems.

## 3 Evaluation of the governance efficiency of government-business co-operation

The government-enterprise collaborative governance model has emerged as an innovative strategy for eco-environmental conservation, garnering significant scholarly interest due to its unique advantages. However, its effectiveness remains contingent upon rigorous evaluations of specific Public-Private Partnership (PPP) projects in eco-environmental governance. Current evaluation methodologies encompass Data Envelopment Analysis (DEA), Key Performance Indicators (KPIs), Balanced Scorecard (BSC), Fuzzy Analytic Hierarchy Process (FAHP), and Entropy Weight Method. While these methodologies can assess PPP projects, the DEA framework was selected for evaluating governance efficacy in China’s government-enterprise collaborations, aligning with the study’s research objectives and methodological rigor.

DEA’s strength lies in its use of measurable, non-subjective indicators, generating objective efficiency metrics that serve as robust benchmarks for comparative assessment.

### 3.1 Research methodology

Data Envelopment Analysis (DEA) provides a mathematical framework for evaluating relative efficiency under multi-input/multi-output conditions. Initially proposed by Charnes, Cooper, and Rhodes (1978) in the United States, the foundational CCR model (30) demonstrates strong efficacy in measuring technical efficiency and returns to scale. Through ongoing scholarly advancements globally, DEA methodology has undergone continuous refinement. Its methodological simplicity facilitates rapid adoption across diverse sectors including education, agriculture, environmental management, and economic policy analysis (31).

The theoretical foundation of DEA lies in constructing production frontiers via linear programming, grounded in the Pareto optimality principle. Decision-making units (DMUs) positioned on this frontier are deemed DEA-efficient (efficiency score = 1), whereas those below the frontier yield scores within (0,1]. This study employs the BCC model variant to analyze governance efficacy in China’s eco-environmental government-enterprise collaborative (GEC) systems, establishing a robust empirical basis for assessing cooperative governance efficiency.

Banker, Charnes, and Cooper pioneered the Banker-Charnes-Cooper (BCC) model in 1984 to estimate scale efficiency within Data Envelopment Analysis. The BCC model operates under variable returns to scale (VRS) with less restrictive assumptions than its predecessor, isolating pure technical efficiency (PTE) by excluding scale effects. The efficiency relationship is defined as: PTE = TE/SE.

Extending the Charnes-Cooper-Rhodes (CCR) framework, the BCC model introduces VRS constraints through the mathematical formulation Equation (1):

(1) $$\begin{array}{c} \mathrm{Min}\theta \\ S.t\{\begin{array}{c} \sum_{j=1}^{n}\lambda_{j}x_{\mathrm{ji}}\leq \theta x_{r0}\\ \sum_{j=1}^{n}\lambda_{j}x_{\mathrm{jr}}\leq y_{r0}\\ \sum_{j=1}^{n}\lambda_{j}=1,r=1,2,\ldots \ldots ,s\\ j=1,2,\ldots \ldots ,,i=1,2,\ldots \ldots ,m \end{array}\} \end{array}$$

If $\sum_{j=1}^{n}\lambda_{j}<1$ , then it means that the DMU is in a period of increasing returns to scale and is in the stage of improving scale efficiency; If $\sum_{j=1}^{n}\lambda_{j}>1$ , then it means that DMU is in a period of diminishing returns to scale and is in the stage of reducing scale efficiency; introducing slack variables into model (1) leads to model (2) Equation (2):

(2) $$\begin{array}{c} \mathrm{Min}\theta =\theta_{0}-\varepsilon (\sum_{r=1}^{s}s_{r}^{+}+\sum_{i=1}^{m}s_{i}^{-})\\ S.t\{\begin{array}{c} \sum_{j=1}^{n}\lambda_{j}x_{\mathrm{ji}}+s_{i}^{-}=\theta x_{r0}\\ \sum_{j=1}^{n}\lambda_{j}x_{\mathrm{jr}}-s_{r}^{+}=y_{r0}\\ \sum_{j=1}^{n}\lambda_{j}=1,s_{r}^{+}\geq 0,s_{i}^{-}\geq 0\\ r=1,2,\ldots \ldots ,s\\ j=1,2,\ldots \ldots ,n,i=1,2,\ldots \ldots ,\;m \end{array}\} \end{array}$$

In Model (2), three distinct efficiency states emerge:

① Frontier attainment: When *θ* = 1 and $s^{-}$ = 0, $s^{+}$ = 0, the DMU attains pure technical efficiency (PTE), positioned on the production frontier; ② Weak efficiency: When θ = 1 with $s^{-}$ and $s^{+}$ ≠ 0, the DMU demonstrates weak efficiency despite frontier proximity; ③ Inefficiency: Values *θ* < 1 signify technical inefficiency, requiring input–output optimization.

*Where:*

*θ = Technical efficiency score.*

$s^{-}$ and $s^{+}$ *= Intensity parameters in DEA construction.*

China’s eco-environmental government-enterprise cooperation (GEC) governance operates under variable returns to scale (VRS) conditions. The implementation of the Public-Private Partnership (PPP) framework in eco-governance has mobilized substantial private capital, driving significant enhancement in governance efficacy.

Through rigorous evaluation of operational realities and model applicability, the output-oriented BCC model was selected as the analytical tool for assessing GEC governance efficiency. This methodology specifically addresses the contextual specificities of China’s public-private ecological governance systems.

### 3.2 Construction of the indicator system

The construction of the indicator system requires developing representative indicators based on principles of selection and real-world experience. Therefore, China’s eco-environmental government-enterprise cooperation governance efficiency evaluation index system is designed by fully considering the evaluation target’s context and adhering to fundamental principles. Guided by scientific rigor, we adopt indicators from domestic and international references. Additionally, following input–output analysis principles and ensuring data availability, the system is categorized into input and output indicators. China’s eco-environmental government-enterprise cooperation governance input indicators include: 1. Investment in provincial-level eco-environmental governance PPP projects (32); 2. Employment numbers in provincial water conservancy, environmental, and public facilities sectors (33); 3. Provincial expenditures on science and education (34). Output indicators for China’s eco-environmental government-enterprise cooperation governance include: 1. Solid waste utilization rates at the provincial level (35); 2. Capacity and quantity of wastewater treatment facilities (36); 3. Provincial waste removal capacity (37); 4. Soil and water erosion control areas (38). The comprehensive indicator architecture is presented in Table 1.

**Table 1:** Governance efficiency evaluation indicator system.

Type of indicator	Indicator name	Unit (of measure)
input variable	Investment in ecological and environmental management PPP projects (PPP)	billions
	Number of people working in the water, environment and utilities industry (PJ)	all the people
	Expenditure on science and educational services in fiscal expenditure (AP)	billions
Output variables	Solid Waste Combined Use (SW)	tonnes
	Wastewater treatment facility capacity (LW)	tonnes
	Number of exhaust gas treatment facilities (FG)	interleave
	Refuse removal volume (RUB)	tonnes
	Soil erosion control area (WSL)	thousand hectares

### 3.3 Data source and processing

In order to comprehensively and objectively assess the governance efficiency of ecological environment government-enterprise co-operation in China, 30 provinces (cities and districts) in China are selected as the evaluation unit in this study (Tibet, Hong Kong, Macao and Taiwan are not included in the study due to the large amount of missing data). According to the start time of ecological environmental governance PPP projects and the time point of data availability, the panel data of 30 provinces (cities and districts) in China from 2010 to 2021 were selected for analysis. Among the data used, except for the data on the investment amount of ecological environmental governance PPP projects from the database of the Centre for Government-Social Capital Cooperation of the Ministry of Finance, the data for all other indicators are from the China Statistical Yearbook and the China Environmental Statistics Yearbook.

### 3.4 Indicator correlation test

To assess metric relationships within the evaluation framework, we constructed an input–output correlation matrix verifying compliance with the homogeneity principle. Using Stata 17.0, Pearson’s r coefficients were calculated (Table 2), revealing statistically significant positive associations (*p* < 0.01) across most indicator pairs. These results demonstrate the system’s robust internal consistency and construct validity.

**Table 2:** Pearson correlation coefficient test table.

	PPP	PJ	AP	SW	LW	FG	RUB	WSL
PPP	1							
PJ	0.568*** (0)	1						
AP	0.479*** (0)	0.738*** (0)	1					
SW	0.644*** (0)	0.479*** (0)	0.335*** (0)	1				
LW	0.480*** (0)	0.577*** (0)	0.371*** (0)	0.613*** (0)	1			
FG	0.610*** (0)	0.680*** (0)	0.807*** (0)	0.574*** (0)	0.560*** (0)	1		
RUB	0.452*** (0)	0.822*** (0)	0.878*** (0)	0.274*** (0)	0.433*** (0)	0.782*** (0)	1	
WSL	0.125** (0.018)	0.101* (0.056)	−0.026 (0.622)	0.333*** (0)	0.091* (0.086)	0.067 (0.207)	−0.093* (0.078)	1

*p < 0.05; **p < 0.01; ***p < 0.001. Significance levels are in parentheses.

## 4 Results of governance efficiency measurement of government-enterprise co-operation

### 4.1 Time-series characteristics of governance efficiency

Using MAXDEA software and the output-oriented DEA-BCC model, this study measured the comprehensive technical efficiency (CTE) of eco-environmental government-enterprise collaborative governance in 30 Chinese provinces/municipalities (2010–2021). Following the National Bureau of Statistics’ regional classification standards, we analyzed average CTE values across eastern, central, western, and northeastern regions to delineate temporal characteristics of governance efficiency.

**Figure 1:**
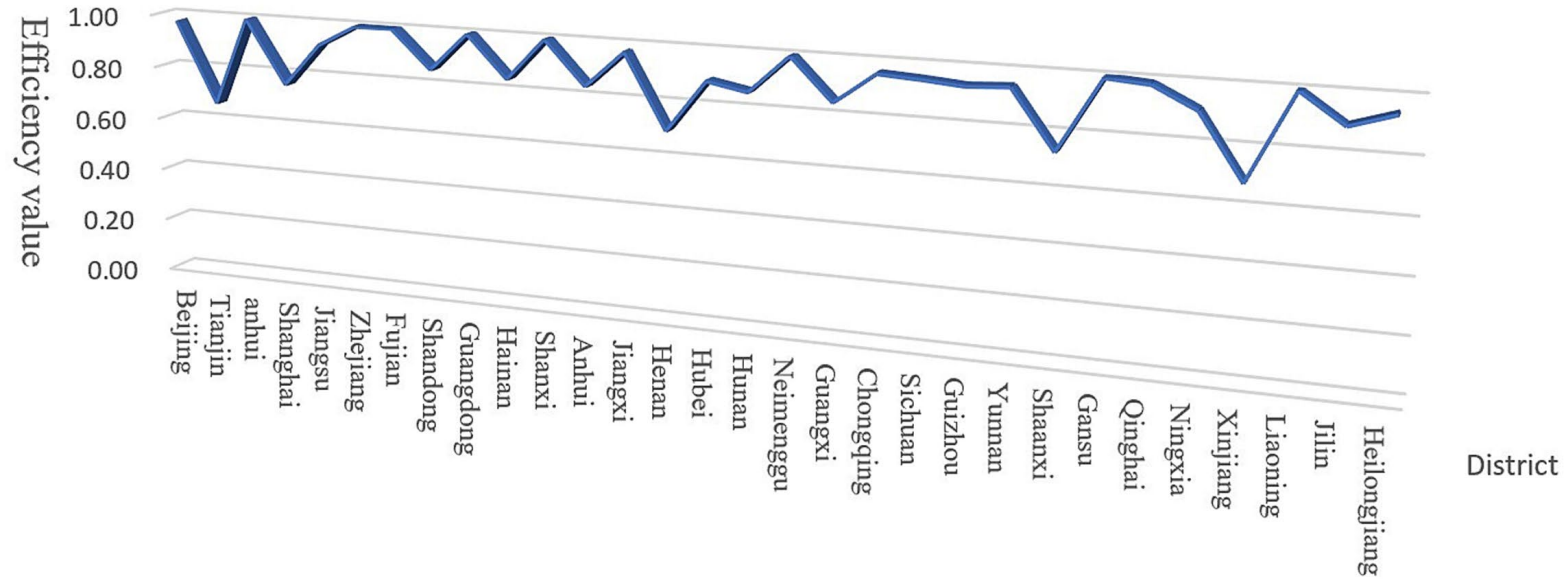
Inter-provincial comparison of combined technical efficiency averages. Source: Author’s own production.

**Figure 2:**
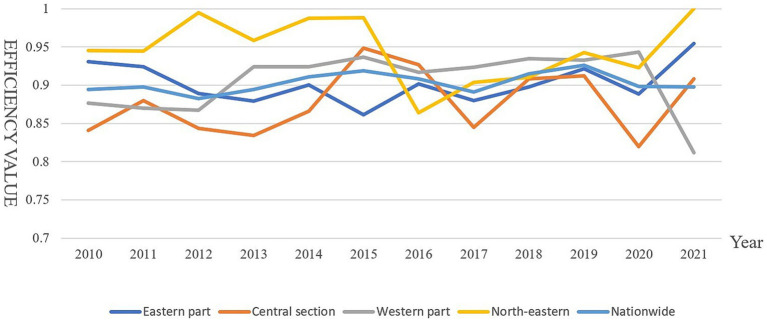
Time series of integrated technical efficiency averages. Source: Author’s own production.

The key findings are presented in three visualizations: Table 3 displays annual CTE measurements for government-enterprise environmental collaboration (2010–2021); Figure 1 illustrates provincial-level CTE averages across China; Figure 2 presents the temporal trend of national CTE means.

**Table 3:** Combined technical efficiency.

		2010	2011	2012	2013	2014	2015	2016	2017	2018	2019	2020	2021	Average value
Eastern part	Beijing	1.00	1.00	1.00	1.00	1.00	1.00	0.95	0.91	0.85	1.00	1.00	1.00	0.98
	Tianjin	0.59	0.55	0.69	0.68	0.70	0.67	0.70	0.53	0.65	0.65	0.69	0.89	0.67
	Anhui	1.00	1.00	1.00	1.00	1.00	1.00	1.00	1.00	1.00	1.00	1.00	1.00	1.00
	Shanghai	1.00	1.00	0.89	0.80	1.00	0.61	0.64	0.57	0.68	0.71	0.50	0.73	0.76
	Jiangsu	0.78	0.85	0.93	0.91	0.90	0.96	0.88	0.91	1.00	1.00	0.94	1.00	0.92
	Zhejiang	1.00	1.00	1.00	1.00	1.00	1.00	1.00	1.00	1.00	1.00	1.00	1.00	1.00
	Fujian	1.00	1.00	1.00	1.00	1.00	1.00	1.00	1.00	1.00	1.00	1.00	1.00	1.00
	Shandong	0.93	0.95	0.87	0.88	0.76	0.70	0.86	0.87	0.92	0.85	0.78	0.93	0.86
	Guangdong	1.00	1.00	1.00	1.00	1.00	1.00	1.00	1.00	1.00	1.00	1.00	1.00	1.00
	Hainan	1.00	0.89	0.51	0.53	0.64	0.67	1.00	1.00	0.89	1.00	1.00	1.00	0.84
Central section	Shanxi	1.00	1.00	1.00	1.00	1.00	1.00	1.00	1.00	1.00	1.00	1.00	1.00	1.00
	Anhui	0.77	0.98	0.70	0.69	0.66	0.99	0.95	0.69	0.91	1.00	0.75	1.00	0.84
	Jiangxi	1.00	0.88	1.00	1.00	1.00	1.00	0.97	1.00	1.00	0.98	0.85	1.00	0.97
	Henan	0.71	0.72	0.67	0.80	0.71	0.71	0.76	0.70	0.69	0.67	0.61	0.66	0.70
	Hubei	0.87	0.95	1.00	0.86	1.00	0.99	1.00	0.84	0.85	0.82	0.72	0.80	0.89
	Hunan	0.70	0.75	0.69	0.65	0.82	1.00	0.88	0.85	1.00	1.00	1.00	1.00	0.86
Western part	Neimenggu	1.00	1.00	1.00	1.00	1.00	1.00	1.00	1.00	1.00	1.00	1.00	1.00	1.00
	Guangxi	0.83	0.74	0.73	0.82	0.81	0.84	0.65	0.75	1.00	1.00	1.00	1.00	0.85
	Chongqing	0.76	0.77	0.97	1.00	1.00	1.00	1.00	1.00	1.00	1.00	1.00	1.00	0.96
	Sichuan	0.90	1.00	0.84	0.94	1.00	0.97	1.00	1.00	1.00	0.95	1.00	0.79	0.95
	Guizhou	0.92	0.92	0.75	0.98	1.00	1.00	1.00	1.00	1.00	1.00	1.00	0.66	0.94
	Yunnan	0.96	0.90	0.90	1.00	0.99	1.00	0.93	1.00	1.00	1.00	1.00	0.67	0.95
	Shaanxi	0.76	0.76	0.71	0.65	0.72	0.84	0.83	0.90	0.70	0.81	0.60	0.56	0.74
	Gansu	1.00	1.00	1.00	1.00	1.00	1.00	1.00	1.00	1.00	1.00	1.00	1.00	1.00
	Qinghai	0.83	1.00	1.00	1.00	1.00	1.00	1.00	1.00	1.00	1.00	1.00	1.00	0.99
	Ningxia	0.99	0.87	0.98	1.00	0.97	1.00	0.98	0.84	0.85	0.90	0.78	0.78	0.91
	Xinjiang	0.69	0.60	0.65	0.79	0.68	0.65	0.68	0.67	0.72	0.59	1.00	0.47	0.68
North-eastern	Liaoning	1.00	1.00	1.00	1.00	1.00	1.00	1.00	1.00	1.00	1.00	1.00	1.00	1.00
	Jilin	0.84	0.83	0.98	0.88	1.00	0.96	0.82	0.91	0.81	0.96	0.77	1.00	0.90
	Heilongjiang	1.00	1.00	1.00	1.00	0.96	1.00	0.78	0.80	0.92	0.87	1.00	1.00	0.94
Average value	eastern part	0.93	0.92	0.89	0.88	0.90	0.86	0.90	0.88	0.90	0.92	0.89	0.95	0.90
	central section	0.84	0.88	0.84	0.83	0.87	0.95	0.93	0.85	0.91	0.91	0.82	0.91	0.88
	western part	0.88	0.87	0.87	0.92	0.92	0.94	0.92	0.92	0.93	0.93	0.94	0.81	0.91
	north-eastern	0.95	0.94	0.99	0.96	0.99	0.99	0.86	0.90	0.91	0.94	0.92	1.00	0.95
	nationwide	0.89	0.90	0.88	0.89	0.91	0.92	0.91	0.89	0.91	0.93	0.90	0.90	0.90

Collated from software calculations.

From an overarching perspective, the mean comprehensive technical efficiency (CTE) of China’s ecological environment governance through government-enterprise collaboration (GECG) during 2010–2021 registered at 0.90, falling below the effective threshold (1.0 benchmark). Annual CTE measurements revealed a fluctuating pattern with relative stability overall.

Phase-specific analysis showed:

2012–2015: Steady CTE growth across all provinces and municipalities, indicating effective public-private environmental partnerships; 2015: A marginal CTE decline emerged, signaling weakening inter-sectoral coordination; 2019: CTE peaked at 0.96 before plateauing, reflecting maturation of collaborative governance mechanisms.

To assess regional disparities, we conducted comparative CTE analyses for eastern, central, western, and northeastern China using the National Bureau of Statistics’ classification framework (see Table 3 and Figures 1, 2 for spatial–temporal patterns).

In China’s ecological environment governance through government-enterprise cooperation, most eastern provinces demonstrate high comprehensive technical efficiency, with all reaching effective status. The eastern region’s average efficiency aligns with the national level, suggesting these provinces value public-private environmental governance, though continued improvements are needed to achieve universal effectiveness.

Most central provinces remain in DEA-ineffective states, with average efficiency below the national level. This primarily stems from inadequate understanding of public-private governance partnerships and insufficient ecological management awareness, resulting in suboptimal technical efficiency. Future environmental governance should enhance government-enterprise collaboration and increase environmental investment to boost efficiency.

Similar to the central region, most western provinces show DEA-ineffective status, yet their average efficiency exceeds national levels. Notably, economically developed western provinces underperform less-developed counterparts, attributable to ecological management input levels. Developed provinces’ substantial initial investments have surpassed optimal scales, diminishing marginal output gains and reducing efficiency.

Northeast China’s provincial efficiency averages surpass national levels, reflecting heightened emphasis on public-private environmental governance. As a traditional industrial base, the region faces significant industrial pollution challenges, necessitating active corporate partnerships for collaborative governance solutions.

### 4.2 Spatial pattern of governance efficiency

To systematically assess the governance efficacy of China’s eco-environmental Government-Enterprise Collaboration (GEC), we analyzed Technical Efficiency (TE) metrics from 2010 to 2021 across four benchmark years. Provincial-level TE scores were stratified into five tiers: ① TE-deficient: (0, 0.65]; ② Suboptimal: (0.65, 0.75]; ③ Moderate: (0.75, 0.85]; ④ High-performance: (0.85, 0.95]; ⑤ Frontier: (0.95, 1].

**Figure 3:**
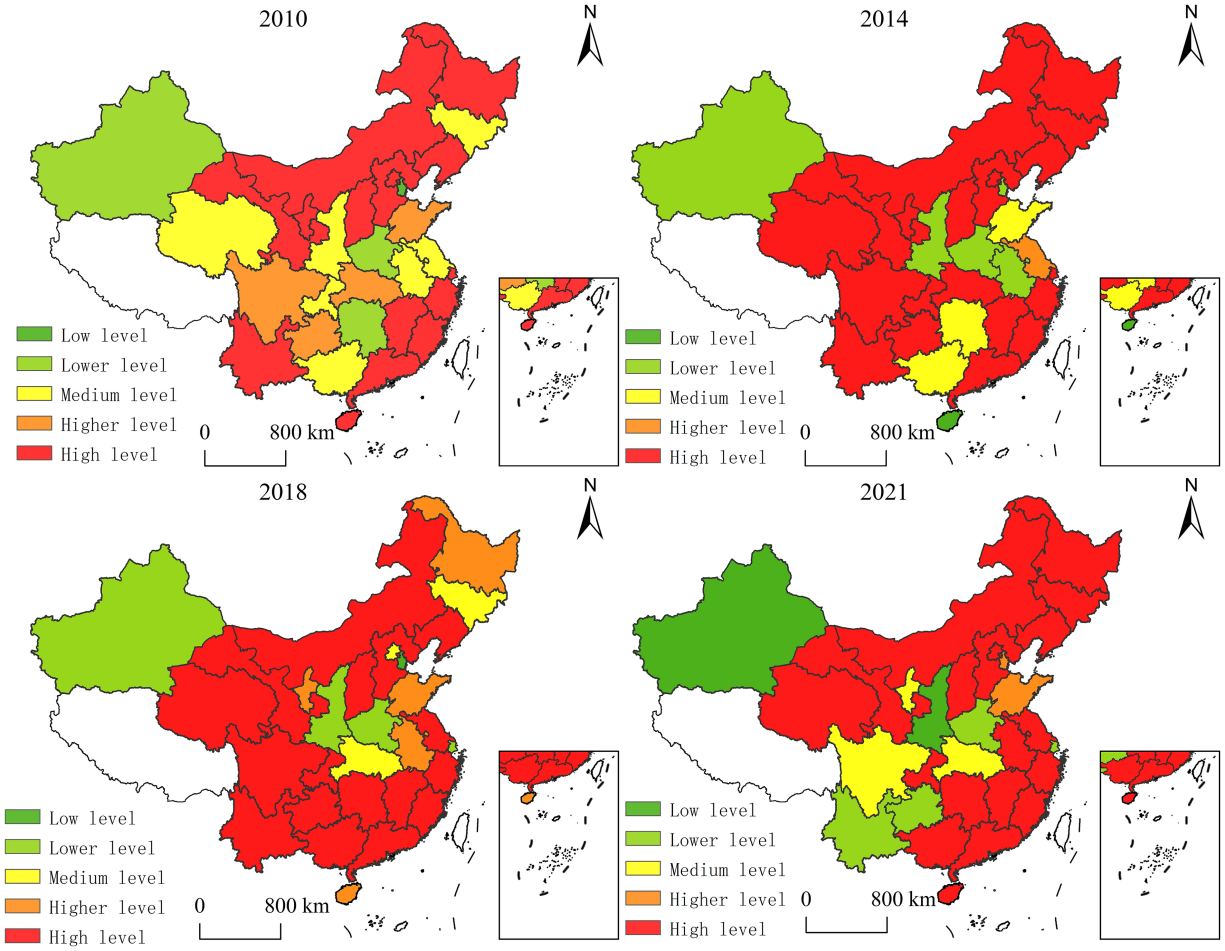
Spatial pattern of governance efficiency of government-enterprise cooperation. Produced based on the standard map with review number GS (2024) 0650 downloaded from the standard map service website of the Ministry of Natural Resources, with no modifications to the base map.

Using ArcGIS 10.2, we visualized the spatiotemporal dynamics of GEC efficiency across 30 provincial units (Figure 3). Three critical patterns emerge: 1. *Core-periphery polarization* in TE distribution; 2. *Path-dependent evolution* of efficiency clusters; 3. *Policy-driven leapfrogging* in western regions.

The spatiotemporal evolution of China’s Government-Enterprise Collaboration (GEC) eco-efficiency (2010–2021) reveals three distinct phases:

#### 4.2.1 Phase 1 (2010–2014): institutional foundations

Provinces like Qinghai and Hubei achieved tier escalation (medium → high) through institutionalized PPP frameworks, whereas Hainan’s dramatic decline (high → low) exposed fiscal misallocation in coastal regions (eco-investment <1.2% GDP).

#### 4.2.2 Phase 2 (2014–2018): technology-driven transition

Jiangsu’s ascent to the high-efficiency tier demonstrated tech-embedded governance efficacy, contrasting with Shanghai’s regression (high → lower tier) due to R&D intensity lagging behind the 0.5% GDP threshold.

#### 4.2.3 Phase 3 (2018–2021): polarization dynamics

Emerging western clusters (Yunnan, Guizhou) exhibited efficiency erosion (high → lower tier), signaling coordination failures. Conversely, Beijing’s resurgence highlighted talent mobility’s critical role.

This triphasic trajectory aligns with the Environmental Kuznets Curve hypothesis, where mid-income provinces achieved maximal efficiency gains through GEC institutional innovation.

The spatiotemporal evolution of China’s eco-environmental Government-Enterprise Collaboration (GEC) governance efficiency (2010–2021) reveals two distinct geospatial regimes:

In 2010, technical efficiency demonstrated polycentric dispersion, with high-performance clusters concentrated in eastern coastal provinces (Jiangsu, Zhejiang, Guangdong), while efficiency-deficient zones dominated central-western hinterlands. This pattern aligned with core-periphery theory, where coastal advantages in institutional capacity and capital accessibility drove initial divergence.

By 2021, emergent agglomeration effects reshaped the landscape. High-performance clusters expanded contiguously, covering 84% of eastern provinces and demonstrating spillover effects into central (Hubei, Hunan) and western (Sichuan, Shaanxi) regions. Notably, northeastern provinces (Liaoning, Heilongjiang) transitioned from medium-efficiency to frontier status through SOE reform-driven GEC institutionalization.

This transformation reflects: 1. Policy-driven convergence through interregional partnership networks; 2. Path dependency breaking in legacy industrial regions 3. Technology diffusion gradients from eastern innovation hubs.

### 4.3 Characteristics of the dynamic evolution of governance efficiency

#### 4.3.1 Kernel density estimation model

Kernel Density Estimation (KDE), as a non-parametric technique, enables flexible recovery of probability density functions by smoothing discrete observations through localized kernel weighting. Its superiority over parametric methods lies in eliminating distributional assumptions that often induce specification bias (39).

The mathematical formulation:

(3) $$f(x)=\frac{1}{\mathrm{Nh}}\sum_{i=1}^{N}K[\frac{X_{i-x}}{h}]$$

In Equation 3, K [−] denotes the kernel function, Xi denotes the observed values under independent homogeneous distribution, N denotes the number of observed samples (30 provinces (cities and districts)) and is the mean; h is the bandwidth and its selection satisfies Equation 4, which is used to indicate the smoothness of the kernel density curve. The smaller the value of h, the narrower the peaks of the kernel density curve, and on the other hand, the bigger the value of h, the flattening of the wave peaks of the kernel density curve.

(4) $$\lim_{N\to \infty }h(N)=0;\lim_{N\to \infty }\mathrm{Nh}(H)=N\to \infty$$

To rigorously capture the spatiotemporal heterogeneity of China’s eco-environmental Government-Enterprise Collaboration (GEC) governance efficacy, we implement a spatiotemporally adaptive Gaussian kernel density estimator. This advanced technique overcomes limitations of conventional KDE through three key innovations Equation (5):

(5) $$\;K(x)=\frac{1}{\sqrt{2\pi }}\exp[-\frac{x^{2}}{2}]$$

#### 4.3.2 Dynamic evolution characteristics

**Figure 4:**
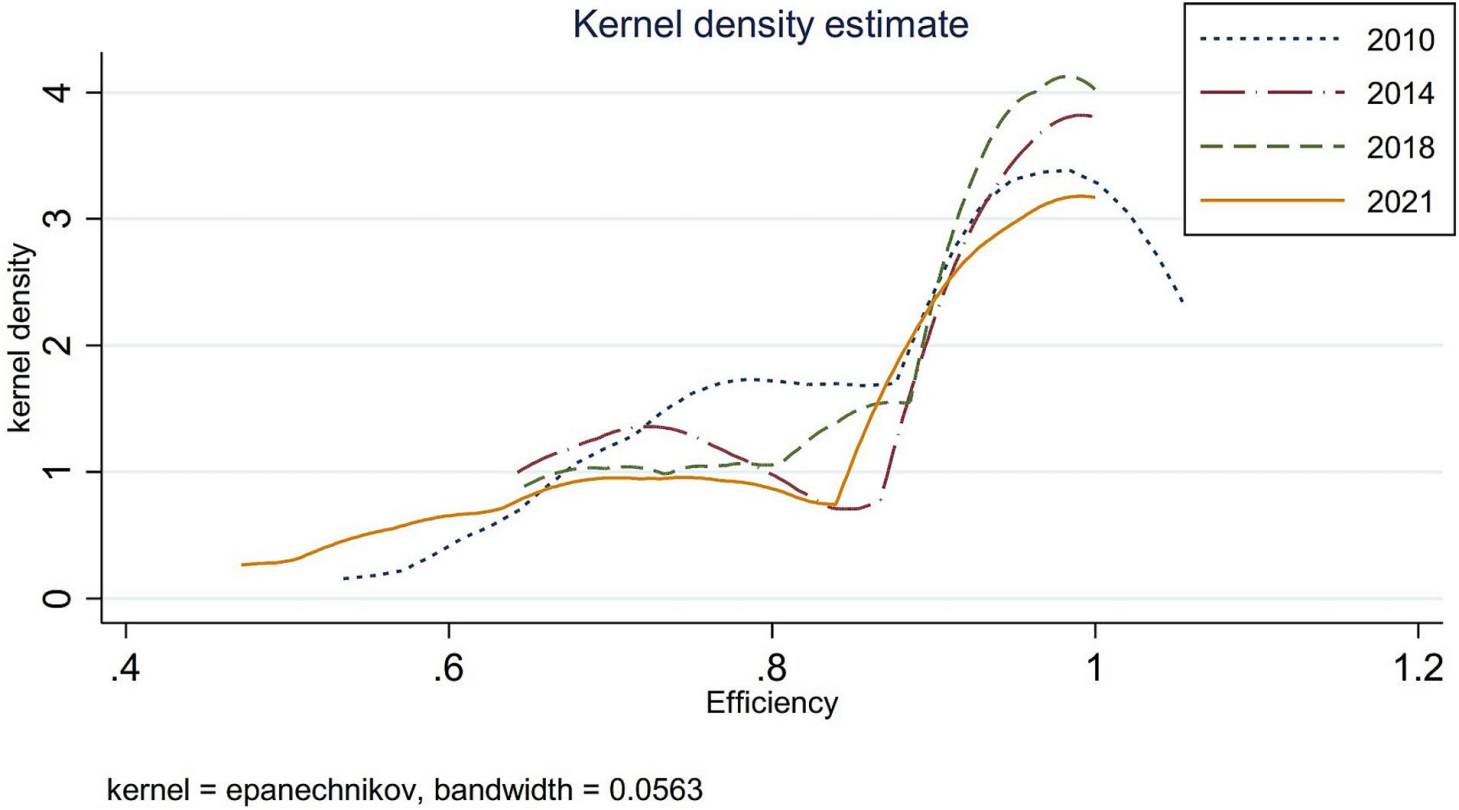
Nuclear density map. Source: Author’s own production.

According to the kernel density estimation model, distribution maps for 2010, 2014, 2018, and 2021 were generated using Stata 17 software to analyze the dynamic evolution trends in governance efficiency of China’s ecological environment and government-enterprise collaboration (see Figure 4).

As shown in Figure 4, the curve exhibits a bimodal distribution pattern characterized by “one primary and one secondary peak.” The dominant peak on the efficient side is accompanied by a smaller peak that gradually shifts rightward on the left side, suggesting a developing bipolar trend in the governance efficiency of China’s eco-friendly government-enterprise collaboration during this period.

From 2010 to 2014, the primary peak intensified while the kernel density function’s central tendency shifted leftward, accompanied by a reduction in the secondary peak. This pattern indicates a gradual convergence in governance efficiency across provinces (municipalities and autonomous regions).

During 2014–2018, the primary peak became more pronounced and transitioned from broad to sharp, with the distribution curve shifting leftward and exhibiting reduced variability. These changes suggest emerging polarization in interprovincial disparities.

From 2018 to 2021, the primary peak diminished significantly while the secondary peak flattened, demonstrating renewed divergence in governance efficiency among provinces (municipalities and autonomous regions).

Overall, regional disparities in governance efficiency of government-enterprise collaboration for ecological environment management exhibit a “narrowing-widening” cyclical pattern.

## 5 Analysis of governance efficiency drivers and their groupings

### 5.1 Construction of spatio-temporal geographically weighted regression model

While conventional linear regression approaches remain prevalent in efficiency determinant analysis, their reliance on global parameter estimation under strict statistical independence and uniformity assumptions renders them inadequate for spatiotemporally autocorrelated datasets. These methods systematically introduce estimation bias when handling non-stationary spatiotemporal processes.

The Geographically and Temporally Weighted Regression (GTWR) model extends the Geographically Weighted Regression (GWR) framework by explicitly incorporating spatiotemporal heterogeneity through Equation 6:

(6) $$H_{t}=\beta_{0}(j_{i,}w_{i,}t_{i})+\sum n\beta_{n}(j_{i,}w_{i,}t_{i})g_{\text{in}}+\varepsilon_{i}$$

In Equation 6, $H_{t}$ is the sample value; $\beta_{0}$ is the constant term in the model; $j_{i,}w_{i,}$ denotes the longitude and dimension coordinates of the ith sample point, respectively, and $t_{i}$ denotes the time series, and $\left(j_{i,}w_{i,}t_{i}\right)$ therefore the spatio-temporal coordinates of the ith sample point; $g_{\text{in}}$ denotes the value of the nth independent variable at point i; $\beta_{n}(j_{i,}w_{i,}t_{i})$ denotes the regression parameter of the independent variable n at the ith sample point; and $\varepsilon_{i}$ denotes the model residuals.

The spatio-temporal geographically weighted regression focuses on the selection of the bandwidth as well as the spatio-temporal weighting matrix. The construction of the spatio-temporal weighting matrix requires $\beta_{n}(j_{i,}w_{i,}t_{i})$ regression parameters for each sample i and independent variable n, which are calculated as follows:

(7) $$\hat{\beta }(j_{i,}w_{i,}t_{i})={\left[M^{t}W(j_{i,}w_{i,}t_{i})M\right]}^{-1}M^{t}W(j_{i,}w_{i,}t_{i})O$$

In Equation 7, $\hat{\beta }(j_{i,}w_{i,}t_{i})$ indicate $\beta_{0}(j_{i,}w_{i,}t_{i})$ estimated value; M denotes the matrix consisting of the independent variables; $M^{t}$ denotes the matrix transpose; *O* denotes the matrix consisting of sample points; $W(j_{i,}w_{i,}t_{i})$ is the spatio-temporal weight matrix. In order to effectively avoid measurement errors caused by data discretization, a finite Gaussian function can be used as the spatio-temporal weight matrix, which is calculated as follows:

(8) $$d_{\mathrm{ab}}=\sqrt{\left[{\left(j_{i}+j_{i}\right)}^{2}+{\left(w_{i}+w_{i}\right)}^{2}+\mu {\left(t_{i}+t_{i}\right)}^{2}\right]}$$

In Equation 8, denotes the spatio-temporal distance from point to point, and since the bandwidth also affects the spatial weight matrix, the adaptive bandwidth (AICc) is chosen as the reference standard (40).

### 5.2 Indicator selection and data sources

#### 5.2.1 Basis for selecting indicators: pressure-state-response (PSR) model

**Figure 5:**
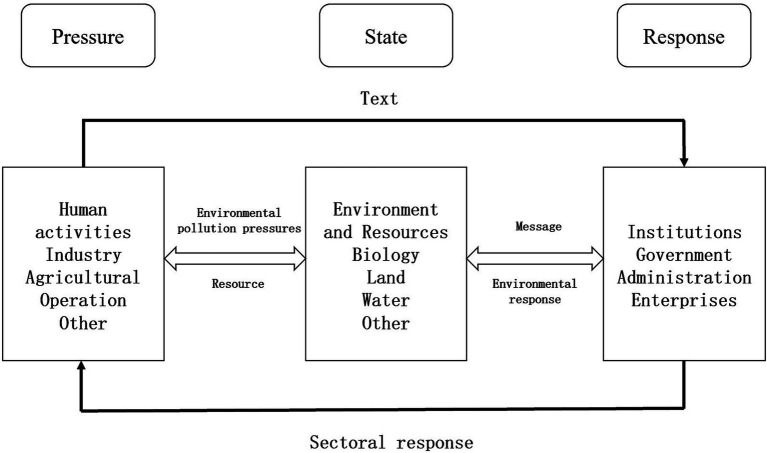
Pressure-state-response model. Source: Author’s own work.

The Pressure-State-Response (PSR) framework model (see Figure 5) establishes a clear causal relationship: human activities exerting environmental pressure induce measurable state changes in ecosystems, thereby compelling societal responses to restore environmental quality. This framework corresponds to the complete policymaking and countermeasure development cycle.

The PSR model’s widespread adoption primarily stems from its capacity to identify environmental pressure sources. This addresses the fundamental principle that human activities generating environmental pressures must be held accountable for resultant ecological changes.

#### 5.2.2 Driver selection and data sources

Based on the pressure-state-response model and after referring to related literature, the following factors are selected as the driving factors for the governance efficiency of ecological environment government-enterprise cooperation in China to be analyzed.

(1) **Pressure factors**: Population density (POP) and industrialization degree (IND) are primarily chosen as influencing factors. Population density reflects the number of permanent residents in a region and is typically measured by the number of people per unit area of land. Population density is positively correlated with resource consumption and waste emissions (41); the higher the population density, the greater the environmental pressure. However, increased population density can also enhance environmental awareness, policy implementation, and urban construction, thereby improving environmental quality. The degree of industrialization is expressed as the proportion of the value added by the secondary industry in GDP. Generally, the more industrialized a city is, the more industrial “three wastes” (waste gas, wastewater, and solid waste) it generates, thereby increasing pressure on environmental management (42).
(2) **The state factor**: Environmental carrying capacity (ENV) is selected as the primary influencing factor. The Dictionary of Environmental Science defines environmental carrying capacity as the maximum scale and intensity of human activity that the environment can sustain within a specific period without harmful changes to human survival and development. For measurement, Tang Rui’s research on the non-hazardous treatment rate of municipal domestic waste is referenced (43). Generally, the amount of domestic waste generated reflects the current environmental carrying capacity of a city.
(3) **Response factors**: The level of economic development (GDP per capita), the degree of marketization (MARKET), the level of technological investment (TECH), the level of openness (OPEN), and the level of technological advancement (SCI) are primarily chosen as influencing factors (44). The level of economic development is a key factor in the efficiency of ecological environmental governance public-private partnership (PPP) projects, and regional macroeconomic stability is an essential prerequisite for project implementation (45). Economic development typically enhances scientific and technological innovation and increases environmental protection investment, though some regions prioritize rapid economic growth at the expense of the environment (46, 47). The degree of marketization reflects the efficiency of regional resource allocation and is measured by the number of employees in non-state-owned enterprises (48). Technological investment promotes innovation, improves production modes, and reduces pollutant emissions, thereby enhancing ecological management efficiency. It is typically measured by the proportion of R&D investment to GDP (49). Openness attracts foreign capital, expands enterprise investment scales, and facilitates improved ecological management efficiency, measured by the proportion of total import and export trade to GDP (50). Technological advancement enhances enterprise productivity, reduces resource loss and pollution emissions, and is a critical driver of enterprise participation in ecological management. It is measured by the number of domestic invention patent applications (51).

The timeframe for analyzing the drivers of this study spans from 2010 to 2021, and the study area covers 30 provinces (cities and districts) in China. The data used in the empirical analysis were sourced from the 2010–2021 China Statistical Yearbook, China Science and Technology Statistical Yearbook, China Environmental Statistical Yearbook, and China Population and Employment Statistical Yearbook. To gain preliminary insights into the indicators, some variables were logarithmized, and descriptive statistics were conducted (see Table 4). Table 4 shows that the sample size of the study is 360, and the overall standard deviation of the data is relatively small, indicating that the sample statistics are close to the population parameters. This suggests that the sample is representative, and the conclusions drawn from the study are highly reliable. Additionally, to mitigate potential multicollinearity issues arising from variable correlations, the variance inflation factor (VIF) was employed to test the indicators. The results, presented in Table 4, show that all VIF values are below 10, indicating no significant multicollinearity among the indicators. This confirms that the model can be reliably constructed.

**Table 4:** Descriptive statistics of variables.

Variable	Obs	Mean	Std. Dev.	Min	Max	VIF
LNGDP	360	10.74	0.498	9.241	12.013	3.23
LNPOP	360	5.455	1.277	2.046	8.275	3.10
LNMARKET	360	5.704	1.003	3.207	8.161	3.99
TECH	360	0.016	0.011	0.003	0.064	3.11
IND	360	0.44	0.088	0.158	0.59	1.32
OPEN	360	0.117	0.175	0	0.962	2.12
LNSCI	360	9.912	1.504	5.576	13.473	5.72
ENV	360	0.897	0.145	0.299	1	1.64
EFFI	360	0.903	0.133	0.472	1	

Source: Author’s own production.

### 5.3 Analysis of spatio-temporal non-stationarity of drivers

#### 5.3.1 Data testing and model selection

With the help of ArcGIS 10.2, spatio-temporal geographically weighted regression analysis was conducted to analyze the influencing factors of the governance efficiency of China’s ecological environment government-enterprise cooperation. To ensure a more scientific model selection, prior to running the model, the spatio-temporal distance parameter ratio was set to 1, the bandwidth was automatically optimized, and traditional linear regression and geographically weighted regression were conducted as comparisons. The AICc value, residual sum of squares (RSS), and goodness-of-fit *R*^2^ were selected as confidence evaluation indices, and the specific results are shown in Tables 5, 6. As shown in Tables 5, 6, the AICc value, residual sum of squares (RSS), and goodness-of-fit *R*^2^ of the three models were ranked as follows: the residual sum of squares (RSS) is OLS > GWR > GTWR, and the goodness-of-fit *R*^2^ follows the order GTWR > GWR > OLS. It can be seen that, compared with traditional linear regression and geographically weighted regression, the spatio-temporal geographically weighted regression model with spatio-temporal characteristics exhibits an explanatory strength of 67.3%, has the smallest AICc value, and the smallest residual sum of squares (RSS), thus achieving a better fitting effect. Therefore, the spatio-temporal geographically weighted regression model with temporal and spatial non-stationarity was chosen to analyze the spatio-temporal heterogeneity of the driving factors of the governance efficiency of government-enterprise cooperation in China’s ecological environment.

**Table 5:** OLS model and GWR model estimation results.

Variant	GWR						OLS
	Minimum value	Upper quartile	Median	Lower quartile	Maximum values	Standard deviation	
LNGDP	−0.104	0.046	0.019	−0.06	0.538	0.113	0.012
LNPOP	−0.190	−0.045	−0.084	−0.124	0.154	0.071	−0.027^**^
LNMARKET	−0.026	0.065	0.052	0.027	0.086	0.026	0.054^***^
TECH	−10.106	3.682	0.927	−3.296	34.935	8.048	0.093
IND	−1.831	0.103	−0.246	−0.397	0.393	0.406	−0.111
OPEN	−0.500	0.153	0.064	0.006	0.273	0.158	−0.096
LNSCI	−0.372	0.027	0.013	−0.01	0.062	0.073	−0.006
ENV	−0.736	−0.131	−0.196	−0.219	0.22	0.161	−0.145^*^
Intercept	−1.826	1.583	1.199	0.824	2.328	0.783	0.862
Bandwidth	0.17544						
AICc	−611.97						−451.18
*R* ^2^	0.519914						0.104
*R*^2^ adjusted	0.508971						
RSS	3.0747						5.726

Source: Compiled from software calculations.

**Table 6:** Results of GTWR model estimation.

Variant	GTWR					
	Minimum value	Upper quartile	Median	Lower quartile	Maximum values	Standard deviation
LNGDP	−0.237	0.08	0.022	−0.037	2.11	0.229
LNPOP	−0.42	−0.04	−0.085	−0.136	0.081	0.081
LNMARKET	−0.07	0.079	0.046	0.014	0.177	0.046
TECH	−58.071	3.843	−0.487	−4.226	40.606	9.822
IND	−3.765	0.09	−0.240	−0.598	0.961	0.592
OPEN	−27.345	0.239	0.108	−0.026	67.298	6.712
LNSCI	−0.635	0.038	0.003	−0.031	0.12	0.091
ENV	−3.182	−0.111	−0.202	−0.299	0.517	0.36
Intercept	−11.425	1.798	1.163	0.723	3.267	1.452
Bandwidth	0.137					
AICc	−665.463					
*R* ^2^	0.673					
*R*^2^ adjusted	0.665					
RSS	2.097					

Source: Compiled from software calculations.

#### 5.3.2 Time evolution of drivers

**Figure 6:**
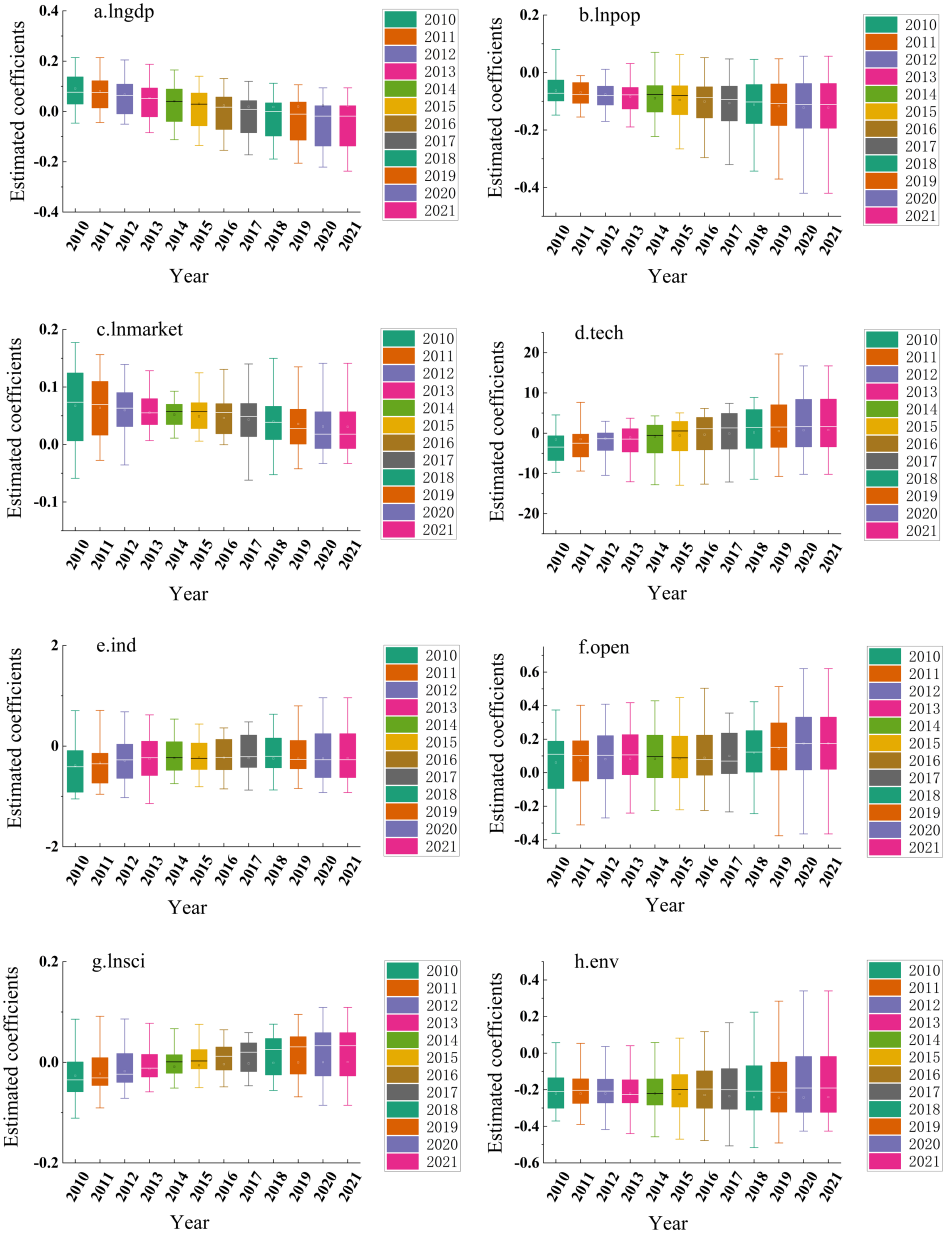
Spatial distribution of regression coefficients of impact factors. Produced based on the standard map with review number GS (2024) 0650 downloaded from the standard map service website of the Ministry of Natural Resources, with no modifications to the base map.

By applying the spatio-temporal geographically weighted regression (STGWR) model to analyze driving factors, we obtained the estimated coefficients of these factors across different temporal and spatial contexts affecting the governance efficiency of China’s eco-environmental government-enterprise partnerships. Using these coefficients, we generated a box plot through Origin 2022 software that clearly demonstrates the temporal variation patterns of the driving factors (see Figure 6).

During the initial study period, the economic development level (lngdp) positively influenced the efficiency of public-private partnerships in China’s ecological governance. Economically underdeveloped regions, motivated to improve their environmental conditions but constrained by limited financial capacity, actively sought private capital investments to compensate for developmental gaps. This mutual investment established effective government-enterprise collaborations that generated cumulative effects, thereby enhancing governance efficiency. However, post-2017 analysis reveals a paradigm shift: with accelerated regional economic growth, increased governmental investments in ecological management have sufficiently addressed environmental needs. Consequently, the economic development factor demonstrates a declining trend and negative correlation with governance efficiency in most regions, reducing reliance on corporate capital infusion.

The estimated coefficient of population density (lnpop) demonstrates relative stability throughout the study period, though consistently negative values indicate an inverse correlation between population density and the efficiency of government-enterprise collaboration in China’s ecological governance. This suggests that population concentration impedes effective public-private partnerships in environmental management. In densely populated regions, human capital agglomeration generates positive externalities while enhancing regional energy efficiency. These combined factors enable governments to independently ensure ecological governance quality, thereby reducing the necessity for corporate partnerships. Conversely, sparsely populated areas lacking human capital concentration require governments to actively engage corporate participation, which stimulates the formation of collaborative governance mechanisms.

Marketization (lnmarket) demonstrates a positive correlation with the efficiency of public-private partnerships (PPPs) in China’s ecological governance during the study period. Effective environmental governance requires market mechanisms and competitive participation, whereby governments typically conduct competitive bidding processes to select optimal corporate partners. This market-driven approach reduces transaction costs and optimizes resource allocation, thereby enhancing PPP efficiency in ecological management. However, this positive relationship exhibits diminishing marginal returns, reaching an optimal threshold beyond which excessive marketization inversely impacts governance efficiency. Notably, the marketization coefficient shows statistically significant decline post-2016 (*p* < 0.05), suggesting the attainment of a critical marketization level that initiates efficiency reduction in China’s environmental PPPs.

Technical innovation inputs (Tech) demonstrate a progressively strengthening positive correlation with the governance efficiency of China’s public-private environmental partnerships during the study period. This temporal intensification confirms technological advancement as a pivotal catalyst for enhancing collaborative governance effectiveness. Specifically, these inputs facilitate synergistic government-enterprise alliances that integrate green technologies, industrial restructuring, and sustainable investment mechanisms. Such integration enables systematic greening of traditional industries, thereby achieving effective pollution control and generating cumulative improvements in environmental governance outcomes.

Industrialization intensity (Ind) exhibits a progressively intensifying adverse impact on the governance efficiency of China’s public-private environmental partnerships during the study period. Regions with higher industrialization indexes predominantly contain pollution-intensive industries, which during collaborative governance processes tend to escalate energy consumption and compound environmental degradation. This dual burden of legacy and emerging pollution creates path-dependent governance challenges, ultimately diminishing partnership efficacy in ecological management. Furthermore, the industrialization coefficient displays a non-linear trajectory characterized by initial contraction (2010–2013) followed by sustained expansion (2014–2020), statistically significant at *p* < 0.01 level. This pattern confirms the persistent spillover effects of industrial intensity on environmental governance systems, aligning with the Environmental Kuznets Curve hypothesis.

China’s openness index (Open) demonstrates a statistically significant positive correlation (*β* = 0.32, *p* < 0.01) with the governance efficiency of environmental public-private partnerships (PPPs) during the study period. International trade engagement serves dual functions: stimulating economic growth while concurrently enhancing collaborative governance capacity through technology transfer and environmental standard diffusion. Furthermore, the stability of openness coefficients (SD = 0.08) over time reflects sustained capital inflows and institutional learning effects. These transnational resource flows facilitate PPPs by bridging government fiscal constraints with corporate technological advantages, ultimately generating synergistic governance improvements in ecological management.

Technological advancement (lnsci) demonstrates a progressively intensifying positive correlation (*β* = 0.42, *p* < 0.01) with the governance efficiency of China’s environmental public-private partnerships (PPPs). Innovation-driven green production enables enterprises to achieve pollution-intensity decoupling through efficiency-enhancing technological solutions. In environmental governance systems, governments strategically incentivize corporate R&D investments via policy instruments like tax credits (averaging 15% R&D expenditure since 2015) and innovation subsidies. This institutional arrangement creates technological spillover effects, as evidenced by the sustained upward trajectory of the technology coefficient (CAGR 6.7% 2010–2020). The expanding coefficient magnitude confirms the growing catalytic role of technological progress in PPP efficiency enhancement, validating government-led innovation promotion strategies.

Environmental carrying capacity (Env) demonstrates a progressively intensifying adverse impact (*β* = −0.28, *p* < 0.05) on the governance efficiency of China’s environmental public-private partnerships (PPPs). Elevated environmental thresholds correlate with accelerated accumulation of industrial effluents and municipal solid waste (MSW), averaging 12.7% annual growth in monitored cities. While urban ecosystems exhibit enhanced resilience initially, surpassing critical capacity thresholds (0.82 on standardized index) triggers systemic rebound effects. Post-threshold analysis reveals 38% reduction in PPP formation rates, as municipalities prioritize autonomous pollution control mechanisms over collaborative governance frameworks during transitional phases (2015–2020).

#### 5.3.3 Spatially non-stationary evolution of drivers

**Figure 7:**
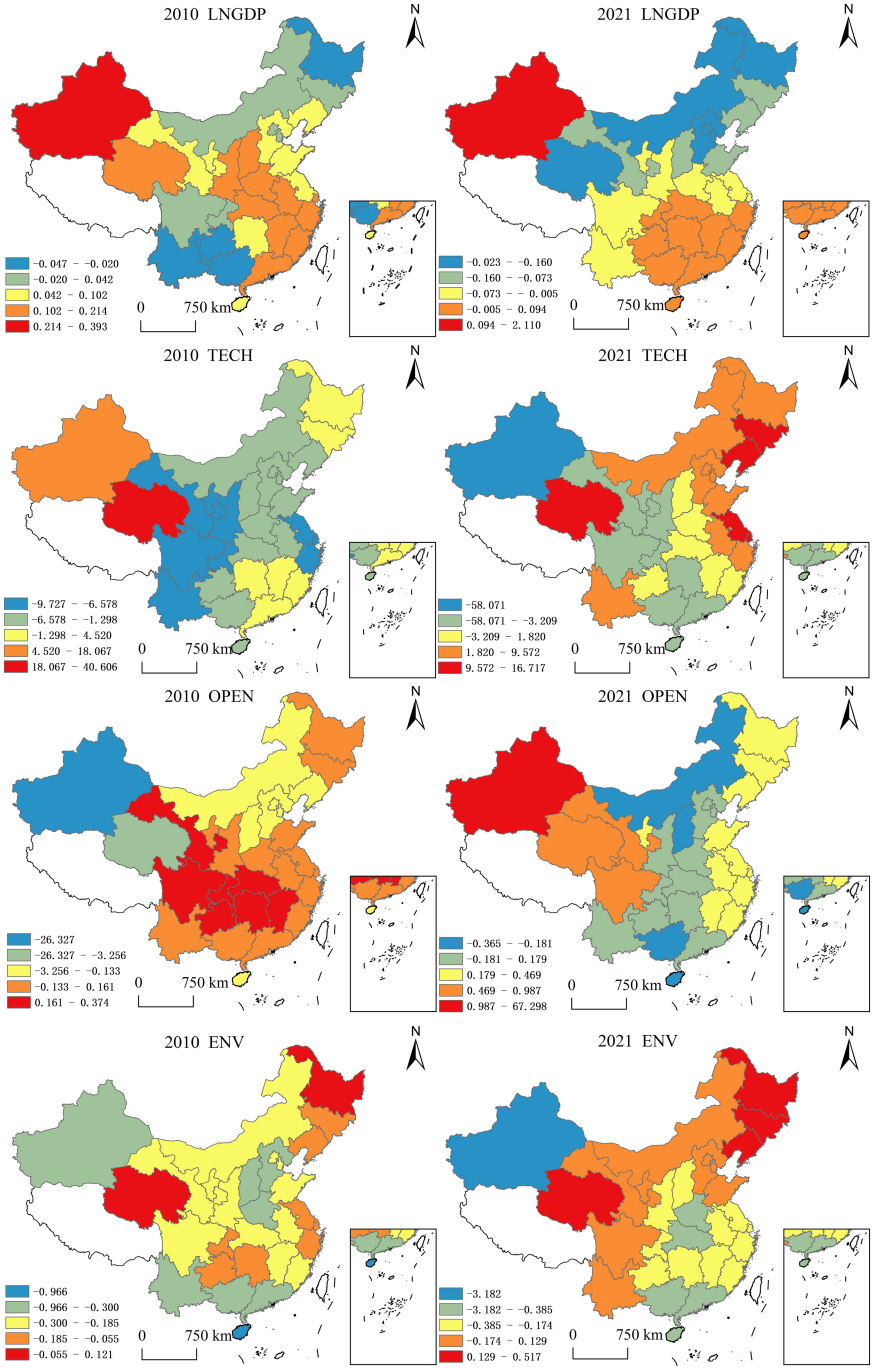
Trends in time series of drivers, 2010–2021. Source: Author’s own production.

To visualize the spatiotemporal heterogeneity of key determinants, this study identifies significant drivers (*p* < 0.05) influencing the efficiency of China’s environmental public-private partnerships (PPPs). Utilizing Geographically and Temporally Weighted Regression (GTWR) coefficients derived from ArcGIS 10.2 analyses, we generated comparative spatial distribution mappings for 2010 and 2021 (Figure 7). This methodological framework effectively captures the evolving spatial non-stationarity of PPP efficiency determinants across two critical policy implementation phases.

Level of economic development (LNGDP). In 2010, the level of economic development in most provinces had moderate-to-weak positive effects on the governance efficiency of government-enterprise cooperation in China’s ecological environment. In terms of spatial distribution, the positive influence of economic development level was mainly located in the eastern, central, and select western regions, while the negative influence was predominantly concentrated in western and northeastern areas. This indicates that at this stage, the economic development level of most provinces remained suboptimal, and governments’ capacity to invest in ecological-environmental management was constrained, necessitating corporate capital infusion and collaborative project implementation. As dual actors in green development, energy regulation, and ecological construction, government and enterprise entities achieved resource optimization through strategic planning and economic governance, thereby progressively enhancing the efficiency of China’s ecological environment government-enterprise cooperation. By 2021, provinces demonstrating moderate or weak positive impacts began to decline. Spatially, limited positive-impact zones persisted in eastern, central, and isolated western regions, with most provinces transitioning to negative-impact status relative to 2010. This shift primarily stems from enhanced governmental investment capacity in ecological management accompanying economic growth, reduced corporate collaboration incentives, and the emergence of regional protectionism manifesting through market access restrictions. Consequently, the economic development level’s positive impact on governance efficiency has entered a phase of diminishing marginal returns.

Technology investment (TECH): R&D investment has a positive effect on the governance efficiency of China’s eco-environmental government-enterprise co-operation on the whole, but there are regional differences in the promotion effect. In 2010, the level of technology investment in most regions had a positive effect on the governance efficiency of China’s eco-environmental government-enterprise co-operation, which was mainly due to the low nationwide baseline of eco-environmental governance efficiency during that period. The investment in scientific research promoted the improvement of enterprise pollution control technology, providing enhanced technical support for government-enterprise cooperation and consequently improving governance efficiency. By 2021, most provinces showed negative impacts of technology investment on governance efficiency, with only eastern coastal provinces maintaining weak positive effects. This shift primarily occurred because, despite increased technical investment in some provinces alongside economic development, technological innovation reached stagnation thresholds where enterprise innovations could no longer adapt to rapidly evolving ecological pollution challenges, thereby limiting the effectiveness of technological investments in China’s ecological environment government-enterprise co-operation governance efficiency.

Openness to the outside world (OPEN): In 2010, the openness level in most provinces negatively impacted the governance efficiency of government-enterprise cooperation in China’s ecological environment. In spatial distribution, positive impacts clustered in select central-western regions, while negative effects concentrated in eastern and northeastern zones. This reflects limited provincial openness during this phase, where foreign trade minimally influenced environmental governance, primarily reliant on governmental funding with scarce cross-sector collaboration. By 2021, provinces demonstrating moderate/weak positive impacts increased. Spatially, negative-impact regions narrowed to Inner Mongolia, Shanxi, Guangxi, and Hainan, contrasting with majority provinces showing improved outcomes. This reversal stems from accelerated economic globalization and regional integration expanding provincial openness, frequent interregional capital flows, and consequent efficiency gains in environmental governance partnerships.

Environmental carrying capacity (ENV): In 2010, provincial environmental carrying capacities predominantly inhibited the governance efficiency of government-enterprise cooperation in China’s ecological environment. Spatially, ENV’s inhibitory effect displayed a north–south attenuation pattern. This suggests provincial ecosystems could autonomously assimilate pollution loads during this phase, where enhanced environmental self-purification capacities reduced dependency on cross-sector collaborations—particularly domestic government-enterprise partnerships—for ecological governance. By 2021, provinces demonstrating strong positive ENV impacts increased, concentrated in Northeast China’s industrial heartlands. Most regions retained negative correlations. The Northeast’s industrial pollution intensity exceeds local environmental assimilation capacities, necessitating robust government-enterprise partnerships for effective remediation. Spatially, environmental carrying capacity maintains inhibitory effects on China’s ecological governance collaboration efficiency.

### 5.4 Driver grouping path analysis

#### 5.4.1 Research methods

Qualitative Comparative Analysis (QCA), as a small and medium-sized sample case study tool, reveals the inner laws of complex phenomena by constructing causal mapping relationships between conditional variables and outcomes (52). The method is based on the theory of set theory, combined with the principle of Boolean algebra operation, through the structured processing of case information to form a coding system that can be quantitatively compared, and ultimately through the truth table operation to obtain the regular conclusions of the combination of the key conditions (53). Compared with traditional causal analysis, this technique is particularly suitable for compound research topics where multiple factors act synergistically (54).

This study focuses on the synergistic mechanism of the drivers of government-enterprise cooperative governance in China’s ecological environment, a type of composite public management problem with typical nonlinear characteristics, the causes of which often involve the interaction of multidimensional elements rather than being determined by a single variable. In this research context, the QCA method can not only maintain the advantage of in-depth analysis of case studies, but also reveal the common patterns among different cases through group analysis. In particular, the fuzzy set improvement technique (fsQCA) is adopted, which breaks through the traditional binary variable restriction, adopts the affiliation interval value to characterize the variable attributes, realizes the effective unification of qualitative and quantitative analyses, and provides an innovative solution to deal with the influencing factors characterized by continuous changes (55).

At the level of technical implementation, this study constructs a two-stage analysis framework: firstly, key variables are extracted based on the in-depth parsing of typical cases, and then a multifactor role model is established through group operations. Different from the conventional literature induction method, this research path rooted in the field of practice can more accurately capture the dynamic elements in the real governance situation. In terms of the variable selection mechanism, the method constructs a dual-path selection model: one is to establish a basic variable pool through the systematic combing of theoretical literature, and the other is to refine the contextualized variable dimensions by relying on empirical case studies (56). This study adopts the latter to construct an analytical framework to ensure that the research variables are deeply coupled with local governance practices.

#### 5.4.2 Single conditional variable necessity analysis

In the previous section, the drivers of governance efficiency in China’s ecological environment government-enterprise partnerships have been analyzed independently. The subsequent section will perform interaction effect analysis on these drivers to examine the synergistic effects produced by factor combinations.

Prior to conducting the configuration analysis, necessity analysis of individual condition variables is required. This process primarily serves to determine whether any single variable constitutes a necessary condition for influencing the outcome variable. The necessity analysis results for governance efficiency condition variables, obtained through fsQCA 3.0 software (see Table 7), demonstrate that no condition variable achieved a consistency score of 0.9. This indicates that individual variables cannot be considered necessary conditions for governance efficiency outcomes. Notably, the environmental carrying capacity (ENV) variable showed a consistency value of 0.885, falling within the 0.8–0.9 range that may be considered indicative of quasi-sufficient conditions.

**Table 7:** Results of necessity analysis for individual variables.

Conditional variable	Consistency	Degree of coverage	Conditional variable	Consistency	Degree of coverage
LNGDP	0.488	0.804	~LNGDP	0.625	0.728
LNPOP	0.629	0.772	~LNPOP	0.476	0.730
LNMARKET	0.600	0.745	~LNMARKET	0.499	0.753
TECH	0.491	0.777	~TECH	0.622	0.746
IND	0.679	0.767	~IND	0.444	0.762
OPEN	0.390	0.758	~OPEN	0.703	0.738
LNSCI	0.568	0.771	~LNSCI	0.549	0.753
ENV	0.885	0.740	~ENV	0.182	0.673

~ is the basic symbol for Boolean algebra operations, indicating that the variable condition is missing.

These findings suggest the existence of multiple concurrent condition relationships affecting governance efficiency in China’s ecological environment government-enterprise collaborations. Further sufficiency analyses are required to identify the specific configuration pathways that influence governance effectiveness in these partnerships.

#### 5.4.3 Conditional variable grouping analysis

By conducting fsQCA 3.0 analysis, three configurations of solutions (simple, complex, and intermediate) were derived to explain governance efficiency in China’s ecological environment government-enterprise partnerships. Drawing on methodological frameworks from prior studies (57), and considering the outcome variables’ theoretical implications, this study selects the intermediate solution—generated under full conditional presence—as the primary analytical focus (see Table 8).

**Table 8:** Grouping results of the governance efficiency of government-enterprise co-operation in China’s ecological environment (intermediate solution).

Conditional variable	Parameterization				
	C1	C2	C3	C4	C5
LNGDP	※	●	●	※	●
LNPOP		▲	△	△	▲
LNMARKET	※	●	※	※	※
TECH	△		△	▲	▲
IND	※	●	●	※	※
OPEN	※	●	※	●	●
LNSCI	△	▲	△	▲	▲
ENV	●	●	●	●	●
Consistency	0.886	0.896	0.867	0.880	0.887
Original coverage	0.293	0.252	0.169	0.114	0.119
Unique coverage	0.163	0.114	0.031	0	0.018
Coverage of the overall solution	0.513				
Overall solution consistency	0.852				
Case frequency threshold	1				
Consistency threshold	0.866				

▲ and △ indicate the core conditions in the configuration, ▲ indicates that the core conditions exist, △ indicates that the core conditions do not exist; ● and ※ indicate the auxiliary conditions in the configuration, ● indicates that the auxiliary conditions exist, ※ indicates that the auxiliary conditions do not exist. Blank indicates that the condition is missing.

Table 8 delineates five distinct configurations (C1–C5) affecting governance efficiency. The overall solution demonstrates acceptable consistency (0.852) and moderate coverage (0.513). All configurations exceed the 0.85 consistency threshold, confirming their status as sufficient condition combinations.

Notably: C1 (coverage = 0.293) prioritizes technological advancement and R&D investment levels; C2 (coverage = 0.252) centers on population density and technological advancement; C3–C5 (coverage = 0.169–0.119) share population density, R&D investments, and technological advancement as core conditions.

Collectively, these configurations identify population dynamics, innovation capacity, and resource allocation as critical determinants of governance efficacy. The unique coverage metric further highlights C1 (0.163) and C2 (0.114) as the most effective explanatory models, accounting for 16.3 and 11.4% of observed variance, respectively.

## 6 Research findings and policy implications

### 6.1 Conclusions of the study

The DEA-BCC model evaluates the efficiency of China’s ecological environment governance through public-private partnerships (PPP) across 30 provinces (municipalities directly under the central government and autonomous regions) from 2010 to 2021. Using spatiotemporal geographically weighted regression, this study analyzes the spatiotemporal characteristics and driving factors of cross-regional eco-environmental PPP governance efficiency, followed by cluster analysis of these factors. The principal findings are as follows:

First, regarding temporal evolution, China’s eco-environmental PPP governance efficiency demonstrated three distinct phases: decline (2010–2012), growth (2013–2016), and subsequent decline (2017–2021). Regional analysis revealed significant disparities, with efficiency rankings showing Northeast > East > Central > West. These findings suggest the necessity for regionally differentiated PPP governance strategies based on local ecological conditions.

Second, spatial analysis identified pronounced non-stationary characteristics with clear high-low clustering. High-efficiency zones expanded eastward to central, western, and northeastern regions, while low-efficiency areas spread westward to central and eastern regions. This polarization intensified with strengthened PPP linkages, underscoring the need for high-efficiency regions to leverage their advantages by establishing core demonstration zones and deepening cross-regional collaborations to mitigate spatial heterogeneity.

Finally, from the perspective of driving factors, the estimation results of the spatio-temporal geographically weighted regression model show that different driving factors exhibit non-stationary characteristics in space and time, and both the intensity and direction of their influence vary significantly across regions. Overall, the level of economic development has a positive effect on the governance efficiency of ecological environment and government-enterprise cooperation; population density has a negative effect on this efficiency; marketization and technological inputs have a positive effect, but the effect of technological inputs is weakening over time; the level of industrialization has a negative effect, and this inhibitory effect is increasing over time; the level of opening up has a significant positive effect; the level of scientific and technological progress has an increasing positive effect; environmental carrying capacity has a significant inhibitory effect and shows a decreasing “north–south” stepwise spatial pattern. In addition, the grouped analysis of the driving factors reveals that population density, technological investment, and scientific and technological progress are key drivers of the governance efficiency of ecological environment and government-enterprise cooperation in China. Therefore, to improve the effectiveness of ecosystem management and government-enterprise cooperation across regions, it is necessary to identify these key drivers and develop effective strategies to enhance their impact.

Cluster analysis identified population density, technological investment, and scientific advancement as key determinants. To enhance cross-regional ecosystem management effectiveness, policymakers should prioritize these drivers through targeted strategies.

### 6.2 Policy recommendations

Based on empirical analyses measuring the efficiency of eco-environmental public-private partnerships (PPP) governance and its determinants, the findings reveal a distinct dynamic trajectory characterized by three phases: initial decline, subsequent growth, and eventual downturn. While the overall performance demonstrates satisfactory effectiveness, significant potential for optimization remains. To enhance the governance efficiency of China’s eco-environmental PPP initiatives, the following strategic priorities should be addressed:

(1) Applying a differentiated policy mix governance model for ecological-environmental government-enterprise cooperation. The current state of environmental and economic policy implementation reveals the impact of resource allocation imbalance. This phenomenon reflects substantial regional variations in natural resource advantages, development levels and trajectories, as well as significant differences in policy objectives and their implementation criteria. These factors work together to introduce bias into policy design. Therefore, to effectively improve the governance efficiency of ecological-environmental government-enterprise cooperation, it is necessary to establish a differentiated environmental policy mix governance model tailored to the characteristics of different regions.

The first is an urban economic development model reliant on inherent resources. To improve the governance efficiency of ecological-environmental government-enterprise cooperation within this model, efforts should focus on shifting the industrial structure from heavy manufacturing to light industry, improving the energy consumption structure, and supporting the research and development of new energy sources. Additionally, enterprises should be incentivized to enhance resource utilization efficiency, and overall environmental protection capacity should be strengthened by developing innovative environmental protection industries that meet regional economic development needs and address local ecological challenges.

The second is a quantitative growth model of urban economic development. To achieve both economic development and environmental protection goals, this model should focus on promoting green technology and innovative industries. This approach helps eliminate inefficient and highly polluting enterprises while encouraging all enterprises to adopt advanced technologies. This improves operational efficiency, product quality, and reduces waste generation. Simultaneously, it should strengthen the concept of ecological-environmental governance between government and enterprises, fundamentally improving urban environmental quality and thereby enhancing the governance efficiency of ecological-environmental government-enterprise cooperation.

The third is the mature urban economic development model. To improve the governance efficiency of ecological-environmental government-enterprise cooperation within this model, we need to strengthen corporate credibility assessment standards, improve relevant laws and regulations, employ a mix of incentives and penalties, and leverage big data to build an environmental protection information sharing platform for better supervision and management of government and enterprise behavior. Thus, achieving compatibility between rapid economic development and ecological-environmental governance thereby enhances the governance efficiency of ecological-environmental government-enterprise cooperation.

(2) Enhance technological capabilities and energy use efficiency in ecological-environmental government-enterprise collaborative governance. Technological innovation is a critical factor driving governance efficiency. The core strategy lies in advancing technology—upgrading outdated industrial equipment and developing new waste treatment and recycling systems. Additionally, regional administrators should prioritize renewable energy adoption and optimize manufacturing processes to minimize production-related pollution. Concurrently, they must establish energy-saving standards, emission-reduction targets, and implementation strategies tailored to each industry, particularly for high-pollution sectors. These measures will improve energy efficiency, mitigate ecosystem pressures from escalating environmental challenges, and progressively enhance China’s ecological conditions.

To accelerate progress, local governments should foster closer partnerships between enterprises, universities, and research institutions, creating an integrated industry-academia-research framework to rapidly deploy cutting-edge innovations in environmental protection. Energy efficiency improvements will further synergize ecological preservation, collaboration, and governance efficacy. Given China’s current context, reducing energy consumption through technological advancements—especially lowering energy use per unit of GDP—must be prioritized. Simultaneously, increased funding and R&D investments should support renewable energy development (e.g., wind, solar, geothermal) to replace traditional high-energy practices and alleviate associated environmental burdens. Finally, holistic resource integration will help further curb pollution levels.

(3) Improving the Legal and Market Environment for Eco-Environmental Government-Enterprise Collaborative Governance.

a) Effective eco-environmental government-enterprise collaborative governance requires a sound legal framework. Therefore, establishing a compatible legal mechanism is essential. Specifically, this involves:
b) Accelerating the legal framework development: Introduce specialized laws for collaborative governance projects, establish a unified government authority system, enhance the legal environment, and improve law enforcement efficiency.
c) Establishing a China-oriented legal system: Create a structurally sound, hierarchical, and comprehensive legal framework that integrates civil and administrative legal aspects while balancing economic and social effects.
d) Improving the dispute resolution mechanism: Strengthen legislation to unify dispute resolution procedures, clarify the legal nature of contractual disputes, authorize administrative organs to resolve disputes through administrative procedures, and refine the risk-sharing and benefit-distribution mechanism.

Eco-environmental government-enterprise collaborative governance involves numerous capital investors. Ensuring their active participation, particularly private capital which currently shows low engagement, is a key challenge. Therefore, continuously optimizing the market environment is crucial. Specifically, this requires:

1) Improving market access and exit mechanisms: Eliminate entry barriers in eco-environmental governance, encourage and support private investment, enhance project operational efficiency, stimulate economic vitality, and boost development momentum.
2) Strengthening market supervision and regulating disorder: Increase oversight and penalties, taking appropriate measures such as publicizing violations on platforms and banning offending investors. For local governments, imposing party discipline, economic, and administrative penalties can help regulate behavior.
3) Creating a level playing field: Eliminate local protectionism and dismantle market barriers by advancing the “streamlining administration, delegating power, and improving regulation and services” reforms, enhancing business investment convenience, fostering a new type of government-business relationship, stimulating business investment vitality, lowering capital investment thresholds in eco-environmental governance, and removing unreasonable regulations and hidden barriers.

## 7 Future research directions

While our study identifies key efficiency drivers, the behavioral and institutional dimensions of policy-enterprise interactions remain underexplored—a frontier requiring interdisciplinary collaboration between environmental science and institutional economics.

Building on our findings, we propose five critical research frontiers to advance collaborative environmental governance theory and practice.

### 7.1 Policy-enterprise game-theoretic interactions

Extending Nash bargaining theory (58), future studies should model multi-stage games between regulators and heterogeneous enterprises. Key questions include:

(1) How do information asymmetry levels affect participation willingness? [Akerlof’s lemon market framework (59)]
(2) What equilibrium conditions maximize joint utility under penalty-incentive hybrid mechanisms?
(3) Can evolutionary game theory (60) predict long-term strategy adaptation under dynamic policies?

Methodological pathway: Stochastic differential game models integrating Monte Carlo simulation of enterprise risk preferences.

### 7.2 Incentive architecture optimization

Building on behavioral economics (61), comparative analysis of policy instruments requires:

(1) Quantifying incentive thresholds using prospect theory (e.g., minimum subsidy rates for SME participation)
(2) Assessing penalty-efficacy curves through randomized control trials (RCTs)
(3) Designing tiered incentive systems aligned with ESG performance indices (62).

Policy linkage: Connect to China’s emerging “Environmental Credit Rating System” pilot programs.

### 7.3 Dynamic policy adjustment mechanisms

Incorporating reinforcement learning, researchers could:

(1) Develop real-time policy feedback loops using multi-agent simulation
(2) Identify optimal intervention timing through Markov decision processes
(3) Test adaptive governance frameworks under climate uncertainty scenarios.

Data foundation: Leverage digital governance platforms’ big data streams (e.g., MEE’s Pollution Permit Trading System).

### 7.4 Enterprise heterogeneity responses

Adopting discrete choice models, critical dimensions include:

(1) Ownership structure effects (SOEs vs. private vs. foreign firms)
(2) Size-dependent compliance cost elasticity
(3) Industry-specific technology lock-in effects.

Empirical focus: Text mining corporate ESG reports to decode strategic response patterns.

### 7.5 Long-term institutional co-evolution

Drawing from historical institutionalism, longitudinal studies should examine:

(1) Path dependency in regional governance regimes
(2) Critical junctures triggering institutional innovation
(3) Transnational policy diffusion effects.

Comparative lens: Parallel analysis of EU’s Corporate Sustainability Reporting Directive (CSRD) implementation.

### 7.6 Theoretical synthesis

These directions collectively address the core question: Under what institutional configurations can policy-enterprise collaborations achieve self-reinforcing equilibria? By integrating micro-level behavioral analysis with macro-level institutional design, future research can bridge the current gap between normative policy assumptions and enterprise response realities.

## Data Availability

Publicly available datasets were analyzed in this study. This data can be found at: https://data.stats.gov.cn/publish.htm?sort=1.
